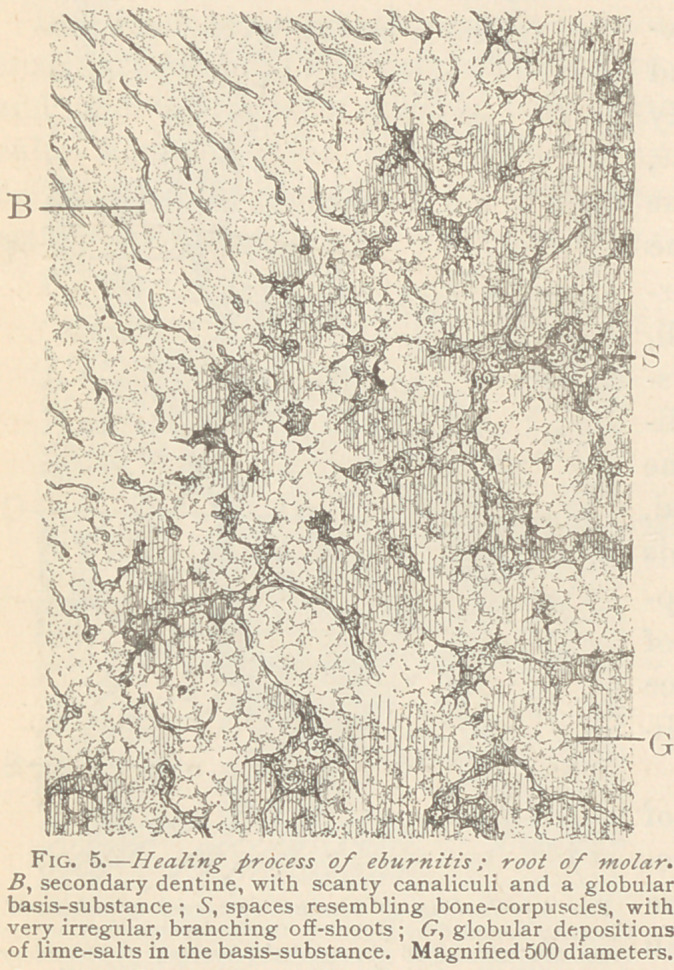# Inflammation of Dentine (Eburnitis)

**Published:** 1886-03

**Authors:** Carl Heitzmann, C. F. W. Bodecker

**Affiliations:** New York; New York


### INFLAMMATION OF DENTINE (EBURNITIS).

BY CARL HEITZMANN, M. D., AND C. F. W. BODECKER, D. D. S., M. D. S.,
NEW YORK.

It is a well-known fact that dentine becomes the seat of an inflammatory reaction by caries, as well as by the gradual destruction of the roots caused by alveolar pyorrhœa, whereby (in the latter instance), first the cementum, and afterward the dentine is destroyed. Another instance of an inflammatory invasion of dentine is observed during pulpitis, where first the secondary and afterward the primary dentine are destroyed by an inflammation of the pulp tissue. Aside from these secondary forms of inflammation, there occurs a primary inflammation in dentine, independent of pulpitis or pericementitis, running its course in the middle of the dentinal tissue, and leading, as all inflammatory processes do, either to a new formation, or to destruction by suppuration. This primary inflammation of dentine is the subject of our paper.

As to the causes of "Eburnitis," as the writers propose to term this process, there are a small number of cases in which nothing can be observed that would account for an inflammatory reaction in the dentine, which, although alive, is far away from the blood vessels throughout. In a few specimens we observed nothing anomalous in the soft parts surrounding the root of the tooth, nor in the pulp tissue. Some preparations exhibited a slight degree of pericementitis, with the characteristic superficial erosions of the cement tissue. In other cases we noticed a layer of secondary dentine, indicative of an increased activity of the pulp tissue, although no pulpitis was visible. In all cases of eburnitis, however, we observed in the dentinal tissue itself a striking feature; an incomplete calcification of the dentine, and a great abundance of interglobular spaces, signifying a malformation and an incomplete calcification of the basis-substance. This accounts for its greater vulnerability and susceptibility to irritative processes. The enamel, in ground specimens, will likewise show abnormalities, such as pigmentation, stratification, or insufficient deposition of lime salts.

Far greater are the number of instances in which traumaticism has led to an inflammation of the dentine. It is evident that any surgical interference, such as burring or excavating, will in an otherwise normal dentine produce irritation sufficient to result in an inflammatory reaction. If, in addition to excavating, a caustic is applied, or a filling inserted, which may exert a certain amount of irritation, inflammation of the dentine must necessarily follow.

We know, from practical experience, that when a caustic in the form of an oxychloride or an oxyphosphate filling is applied to very sensitive dentine, this tissue, in the course of time, will become less sensitive by the partial destruction of the living matter in the dentinal wall of the cavity. The filling material in this manner excites an inflammatory reaction, which in the course of months or years results in a new formation of dentine around the filling, and this is often denser than the original dentine. Thus, in dental practice, we take advantage of this fact, and prior to the introduction of gold, fill very sensitive, or soft teeth, with oxyphosphate or oxychloride of zinc. This hardening, as the most favorable result of traumaticism, is the consequence of an inflammatory reaction of the dentine, known under the term of consolidation. On the other hand, instances are not rare in which the insertion of an oxyphosphate, oxychloride, or a gold filling gives rise to excruciating pain, sometimes even necessitating the removal of the filling. The latter result is due to intense and acute inflammation of the dentine, manifested probably through the medium of the dentinal fibres encroaching upon the pulp tissue. Such unfavorable results are met with mainly in patients whose teeth are badly calcified, or in temporary teeth, in which the amount of living matter is much greater than in the average permanent teeth.

The best known instance of traumaticism applied to dentine is the elephant's tusk, in which a bullet has lodged. The remarkable changes of the dentine in these instances are well known to scientists as well as mechanics. As early as 1798, the great German poet, Goethe,* investigated the subject of diseased ivory from the elephant's tusk, resulting from the impaction of iron or leaden balls; the process appeared to him to be a sort of coagulation (Gerinnung); he also mentions the occurrence of exostosis upon the wall of the pulp cavity, in cases where a ball entered the posterior, weak and hollow portion of the tooth. Cuvier likewise recognized the irregularity in the dental mass around the balls. Richard

---

* The Pathology of the Teeth, Philad., 1872, pp, 188 and 301, *et seq.*

Owen* also noticed the histological changes in elephants' tusks produced by musket balls. On Page 643 we read :

"It is not uncommon to find processes of osteo-dentine, or imperfect bone-like ivory, projecting in a stalactitic form (1) into the interior of the pulp cavity, apparently the consequence of partial inflammation or malformation of the vascular pulp. The musket balls and other foreign bodies which are occasionally found in ivory are immediately surrounded by osteo-dentine in greater or less quantity."

Carl Wedl, in describing the changes in ivory produced by bullets, remarks :

" We are indebted to J. Tomes for the very thorough description of cavities of this nature in two tusks. The dentinal substance in each of the two tusks presented a newly formed cavity, having no connection with the pulp cavity, nor indeed any outlet. * * * By the kindness of Prof. von Schroff, I had the opportunity of examining segments of elephants' tusks which were labelled ulcers. They contain extensive abscess-cavities in the dentine, apparently entirely shut in, of the size of a pigeon's, hen's, even of a goose's egg. * * * If we trace the process of resorption of the ivory from the side of the encroaching osseous tissue, it will be observed that both the main trunks and the lateral branches of the dentinal canals present numerous varicose expansions, while portions of them are transformed into jagged, elongated cavities, or give place to a globular transparent substance. In other portions, large, multiradiating bone-corpuscles have encroached upon the dentine. * * * The chronic inflammation of the periphery of the abscess (Abscesshaut) in these cases, led to the production of solid tissue (osseous and dentinal), both of which must have been developed from cells. But even if we are willing to admit that the blood vessels of the new-formed substance are derived from other pre-existing ones, still the appearance of the new osseous and dentinal substance in the wall of the abscess-cavity continues to be an extraordinary phenomenon, and the assumption in relation to their appearance, that the cell-life of the connective tissue parietes of the abscess is exalted to a differentiation as in embryonic life, is open for discussion, since, indeed, we cannot by any means presuppose that the germs of the formative cells are transported to the part with the blood. * * * The molars of elephants being enclosed within the mouth, are less liable to be penetrated by balls. A case in point, however, is illustrated in Fig. 89, where in a molar an osteo-dentinal mass, inclosing a flattened leaden ball, is interposed between the folds of the enamel on the other side in the substance of the dentine and is in relation with the cement. * * If the three dental substances be traced out, it will be seen

---

* Odontography, London, 1840–1845, p. 646.

that the enamel at a certain distance from the fragment of the ball, and also the dentine, have been displaced by a substance which forms an investment of varying thickness around the fragment of the ball, and when traced further, is found to enter into immediate connection with the cement. Hence the new formation was developed by a proliferation into the enamel and dentine. * * * I endeavored to identify in the dried mass which lines the cavity of an abscess a vascularized connective tissue, from which the blood-vessels, that are prolonged toward the ivory, may possibly have originated, but was unable to come to any satisfactory conclusion in consequence of the marked degeneration of the mass. * * * We find, then, that in the vicinity of these chronic abscesses also a more vigorous reparative tissue is developed in the place of the less vigorous; in other words, an interstitial growth of osseous substance ensues in the vicinity of the cavity of the abscess. * * *

Thomas Bell* reports a case as follows: Mr. S., a medical gentleman, had long been suffering extreme pain in the right side of the lower jaw, apparently produced by the second molar tooth, which, however, had no external marks of disease. After a time, inflammation took place in the periosteum of the root, and the tooth was in a measure loosened. As it now became evident that the cause of the pain, which still continued to the most excruciating degree, was produced by this tooth, it was extracted, and as no diseased appearance was found on its surface, I sawed it asunder at the crown, and found a cavity in the solid bony structure, perfectly circumscribed, the surrounding bone being white, and of a healthy and sound texture. Not the slightest appearance of disease existed in any other part of the tooth, excepting that from the inflammation which had so long existed the membrane had also begun to suppurate. In this case, then, it appears that inflammation had occurred from some local cause in the bone of the tooth; that the vessels of the bone had formed pus, and that absorption had taken place in consequence of its pressure, and formed a cavity for its reception.

Edward Albrecht† also mentions the occurrence of abscesses in the dentine, but believes that they are the result of pulpitis, these cavities being, by a new formation of secondary dentine, separated again from the pulp cavity, although he found the abscess cavities filled with pus.

The present writers have had no chance to study an elephant's tusk immediately after its injury, but the illustrations as given by Carl Wedl are sufficient for the assertion that all the changes in the ivory are produced by an inflammatory reaction around the foreign body driven into it. The result in this instance is exactly the same

---

*The Anatomy, Physiology and Diseases of the Teeth, Philadelphia, 1830, p, 171.

†Die Krankheiten der Zahnpulpa, Berlin, 1858.

as in human dentine in an inflamed condition, caused by foreign bodies.

In order to realize the structural changes in inflammation of the dentine, let us remember that it, like bone, is a living tissue. The analogy between these two tissues is clearly established upon the fact that, in the development of bone tissue, we observe globular territories, the same as in the formation of dentine. In the former instance every territory contains one or more bone corpuscles, with their off-shoots (lacunæ and canaliculi), whereas, in the dentine, the territories are pierced by the canaliculi and their tenants, the dentinal fibres. The basis-substance in both these tissues is traversed by a large amount of living matter, in the shape of a delicate reticulum, the meshes of which are filled with the calcified basis-substance proper. The study of the history of development of bone and dentine reveals a striking similarity in the formation of both of these tissues. The osteoblasts are preliminary formations in developing bone tissue, in the same manner as are the odontoblasts at the periphery of growing dentine. All attempts to explain the development of dentine directly from the odontoblasts have proved unsuccessful, as neither the formation of the basis-substance nor that of the dentinal fibres could ever be brought in accordance with the elongated odontoblasts, whereas, the development of the dentine becomes plain, if we take the ground that the odontoblasts break up into medullary corpuscles, between which the dentinal fibres are formed. If the odontoblasts would calcify directly, we were at a loss to understand the formation of globular territories in fully devoloped dentine. If, on the contrary, we accept the formation of basis-substance from medullary corpuscles, the appearance of globular territories becomes plain. Nothing is required but a group of medullary corpuscles, which are transformed into basis-substance, while the larger threads of living matter, known as Tomes fibres, traverse the rows of medullary corpuscles, taking their course between them. Six years' study of the history of development of the teeth, especially of specimens of the sixth month of intra-uterine life, when dentine begins to form, has led us to the conviction stated above.

Inflammation causes a solution of the lime salts, and afterward a liquefaction of the basis-substance, both in bone and dentinal tissue. The result will be the appearance of globular spaces, or baylike excavations, which, instead of being-filled with basis-substance, exhibit medullary corpuscles, or multinuclear protoplasmic masses, corresponding to the embryonal stage of the inflamed tissue. (See Fig. 1.) The excavations of the dentine are identical with those seen in the process of absorption of the dentine of temporary teeth, and those of secondary dentine in the neighborhood of an inflamed pulp. The diagnosis of primary eburnitis becomes established, not by the appearance of such excavations, but by their presence in the middle of the dentine without any connection with the surface, or the pulp chamber of the tooth.

FIG. 1.—*Eburnitis; neck of tooth.* C, cementum, with shallow excavations at its periphery, caused by pericementitis. D, dentine in the beginning of dissolution of lime-salts; E, protoplasmic masses in globular territories. Magnified 200 diameters.

The earliest feature of eburnitis under the microscope is the appearance in the middle of the dentine of bay-like excavations of varying sizes, and separated from one another by glistening ledges. At first the dentinal canaliculi and their tenants (the Tomes fibres) remain recognizable within the basis-substance. In the next stage only dentinal fibres remain discernible, whereas the contours of the canaliculi are lost, and the basis-substance itself looks irregularly granular. The difference in the refraction of light of these globular spaces, as compared with normal dentine, indicates a dissolution of lime-salts within them. In a still further stage the whole basis-substance is transformed into a granular mass, which is easily stained by an ammoniacal solution of carmine. The globular spaces appear to be filled with multinuclear protoplasmic masses, or with a number of medullary corpuscles, more or less coarsely granular, and flattening each other to some extent. These features, therefore (as mentioned), are identical with those observed in absorption of temporary teeth, the only difference being that the latter process starts from the periphery, whereas eburnitis begins in the middle of the dentine, without any direct connection with the outer surface or the pulp cavity of the tooth, and often at the border of cavities that previously have been filled. (See Fig. 2.)

FIG. 2.—*Eburnitis; crown of molar.* D, beginning dissolution of lime-salts in the middle of dentine; M, medullary corpuscles filling the globular spaces that have arisen from inflamed territories of dentine; C, cavity in the dentine, whose contents had been dragged out. Magnified 500 diameters.

The origin of the globular fields of dissolution in temporary teeth, in some instances, may be questioned, since it is admitted that they are simply the results of the dissolution of the lime-salts, while their filling in with medullary corpuscles may be caused by immigration from without. But in all the specimens of eburnitis which we have examined, no doubt was left as to the primary origin of the spaces and their contents within the dentine, for many of them were isolated, and sometimes far apart from the original seat of inflammation and far from the pulp-chamber as well as the periphery of the the tooth. In connection with eburnitis we frequently have noticed that the crowns of teeth thus affected were surrounded by defective enamel. No doubt can arise that the medullary corpuscles present in these spaces have really grown from dentinal tissue, respectively, from the living matter of the dentinal fibres as well as that of the basis-substance.

In some of the specimens, especially in the central portions of somewhat larger cavities, the medullary tissue, by the process of grinding or cutting, had been removed. Where, however, they had been left in situation, the medullary corpuscles appeared inter-connected by means of delicate off-shoots, representing an embryonal tissue. Or no distinct boundary lines were seen between the medullary corpuscles, the whole filling of a globular territory being a uniformly granular protoplasmic mass, with interspersed nuclei. We cannot deny the possibility that by the breaking apart of these medullary corpuscles pus may be formed in the middle of the dentine, thus representing an abscess independently of the pulp tissue. Wedl and Albrecht take the presence of blood-vessels, presumably grown into the dentine from the pulp, for an absolute necessity in cases of abscess of the dentine. Such a presumption is superfluous, in our opinion, for we assert the dentine to be a living tissue, and as such capable of primary inflammation, nay, suppuration, without a direct co-operation of blood-vessels. We fully realize the possibility that even a so-called pyogenic membrane around the abscess could form without a direct supply of blood-vessels. If others have claimed that an abscess in the dentine must have formed originally in connection with the pulp-tissue, and afterward been separated from the latter, we again deny any such occurrences, since we admit the possibility of the formation of a primary abscess in the dentine, independently of the pulp-tissue.

Far more common than suppuration, however, is the healing process of eburnitis, the results of which may be either a new formation of dentine, closely resembling secondary dentine, or a dentine which is destitute of canaliculi, representing what authors have termed osteo-dentine. In a former publication on "The Distribution of Living Matter in Human Dentine, Cement and Enamel" (Dental Cosmos, 1878), the fact was mentioned that in some teeth certain portions of the dentine are composed of basis-substance only, and devoid of dentinal canaliculi. Later observations have proved that such appearances are due to a very perfect healing of a former inflammatory process. We meet with places in the dentine, more especially in the neighborhood of fillings of some years' standing, wherein the dentine is largely composed of calcified basis-substance, and the dentinal canaliculi are arranged in bundles more or less apart, but of a normal appearance. It is obvious that here the recalcification of the previously inflamed dentine was the most perfect, producing a tissue which is harder and richer in lime salts than the original dentine. For practical purposes, this is the most desirable result obtainable, and it is one of the principal reasons why, in badly calcified teeth, oxyphosphate or oxychloride of zinc should be employed prior to the introduction of a gold filling.

In a second instance, the newly formed tissue closely resembles secondary dentine, with few, irregularly scattered, wavy canaliculi, and large territories of calcified basis-substance, destitute of canaliculi. Sometimes the basis-substance may be uniform in structure, or composed of globular fields or territories, as described in the article on "Secondary Dentine" (Dental Cosmos, 1879.) The tissue as such under the microscope could not be distinguished from secondary dentine; the fact, however, that it was found far from the pulp chamber, in the middle of otherwise normal dentine, proves that it is the result of a previous inflammatory process of the dentine. Here the calcification still attained a high degree of perfection, desirable from the practical standpoint, although morphologically not as perfect as in the first instance. Fig. 3 illustrates this kind of a healing process of eburnitis. Here the cavity, whose soft, medullary contents had been dragged out in the process of grinding, is surrounded by a tissue bearing all characteristics of secondary dentine.

FIG. 3.—*Healing of eburnitis; crown of molar.* *C*, empty cavity; *M*, medullary tissue, at the border of the cavity, partly calcified; *S*, secondary dentine, with scanty and irregularly scattered canaliculi. Magnified 100 diameters.

Another result of eburnitis is the reformation of the basis-substance, composed of small globular masses, between which are visible irregular and widened canaliculi. It is obvious that calcification in this instance is deficient, and consequently the newly formed dentine is less consistent than normal dentine. In one of our specimens, dentine of a temporary tooth, we observed not far apart two spots, one of which shows a large amount of basis-substance, with scanty and narrow canaliculi, whereas another spot was in the condition before described, viz., scantily calcified. The latter portion exhibited well marked globular territories all around a cavity, which probably contained medullary tissue, before subjection to the process of grinding. Some territories contain numerous and wide canaliculi, others scarcely any. Crystals of hæmatoidin indicate the inundation of the inflamed tissue with blood, in the acute stage. As this cavity was near the pulp-chamber, the saturation of the tissue in consequence of a hæmorrhage is explicable. (See Fig. 4.)

FIG. 4.—*Healing of eburnitis; crown of bicuspid. C*, cavity bounded by globular territories between which there are uncalcified interglobular spaces; *S*, secondary dentine; *H*, Crystals of rust brown hæmatoidin. Magnified 500 diameters.

In a fourth instance of eburnitis the healing process is rather poor. There are no new canaliculi formed, but the whole field is traversed by irregular, angular, branching spaces, with numerous radiating off-shoots, bearing a close resemblance to bone corpuscles. The presence of such spaces gave rise to the term "osteo-dentine," applied by previous authors. We would agree with this term if the restriction be added, that in these instances no regular bone-tissue is combined with dentine, but only a tissue resembling bone. The spaces are filled either with medullary corpuscles, or a homogeneous basis-substance, or highly refracting globular masses, the result of a late but rather imperfect deposition of lime salts. The basis-substance between these spaces is likewise composed of globular calcified masses, between which we observe branching off-shoots of the spaces. The periphery of a spot of healed eburnitis may show small spaces scantily scattered in the basis-substance, whereas, the central portions may exhibit very large spaces and comparatively

2

little basis-substance. Fig. 5 illustrates such a termination of eburnitis, partly with a tolerably well developed secondary dentine, partly with a poorly calcified, so-called osteodentine, in which the peripheral zones are decidedly richer in lime-salts than the central ones. Evidently the healing process of eburnitis is the poorest in the last instance, leading, as it were, to the formation of a comparatively soft, brittle and crumbling dentine, lacking the elasticity of the original tissue, and not entitled to the name of dentine proper. From the description given of healed eburnitis around gun-balls in elephants' tusks, it is obvious that the mass, as described as a result of "consolidation" or "coagulation," etc., is such a poorly calcified dentine, or osteo-dentine.

## Figures and Tables

**Fig. 1. f1:**
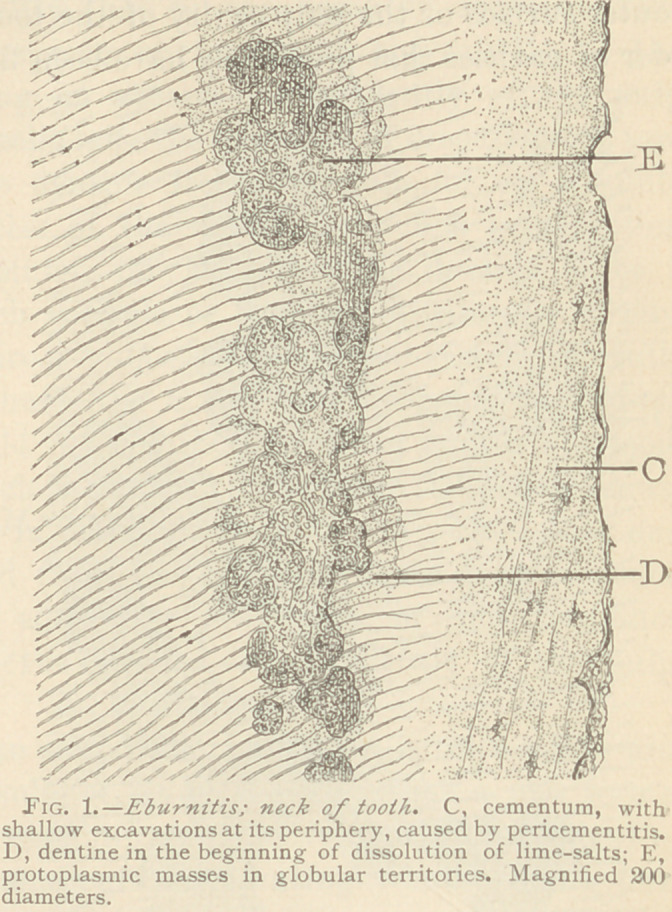


**Fig. 2. f2:**
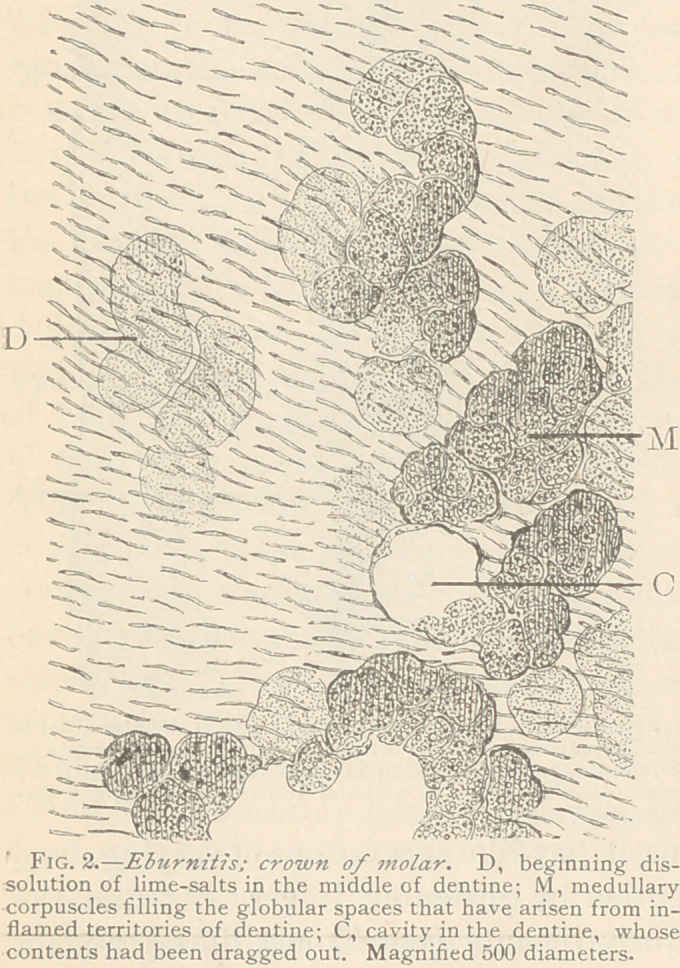


**Fig. 3. f3:**
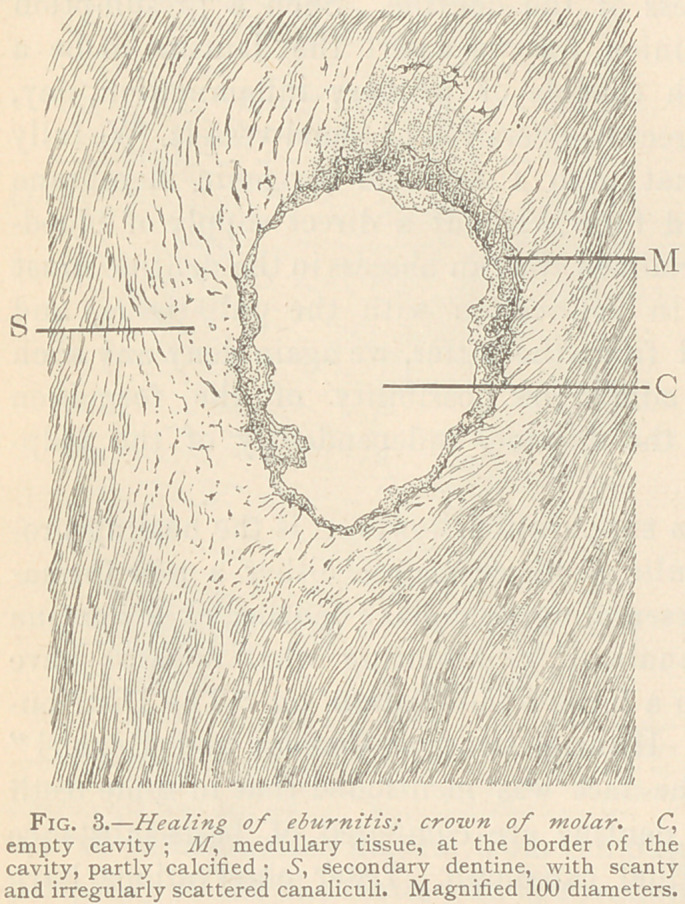


**Fig. 4. f4:**
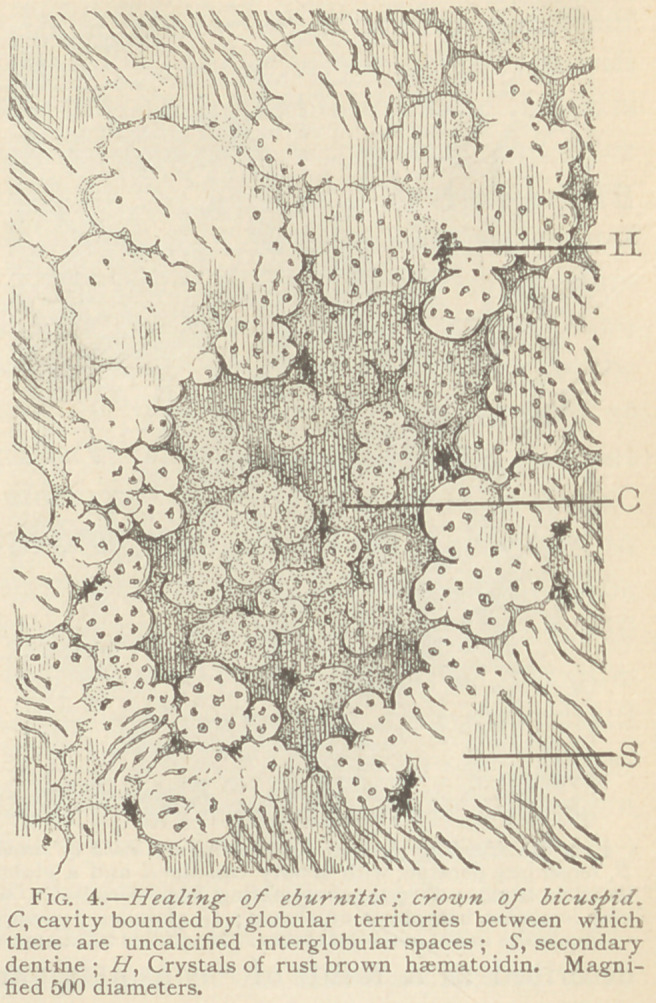


**Fig. 5. f5:**